# The Suggested Scale of Miniumum Salaries

**Published:** 1921-01-15

**Authors:** 


### THE MATRONS' AND SISTERS' DEPARTMENT.

### THE SUGGESTED SCALE OF MINIUMUM SALARIES.

The scale of minimum salaries suggested by the College of Nursing in April 1919 was regarded on its first issue as somewhat revolutionary in tone. It marked, in fact, an advance on prevailing rates, which caused many managers to open their eyes very wide. Take, for example, the rate of £350 for the matron's salary in an institution containing over 500 beds. The Committee had evidence before them that in one such institution the actual rate paid was £80 a year, while the average in the institutions making returns nowhere rose above £310; in London Poor-law infirmaries (eleven in number) it stood at £126, and in provincial Poor-law infirmaries (eleven in number) at £156. We recall these figures because our opinion has never varied that among all the cases of under-pay for which the nursing profession is conspicuous, the case of the matrons is most glaring. When the rigorous system of the survival of the fittest through which matrons pass on the way to their giddy eminence be fully considered it is nothing short of a scandal that no better reward for talent and experience was open to them in the past.

The effect produced by the issue of the College circular of 1919 was immediate and lasting. We are not among those who denounce the managers of institutions as intentional exploiters of nurses. They are men who are administering funds contributed by the public, either of free will or compulsorily through the rates, and their main canon in fixing salaries has been the rough-and-ready principle of not paying more than other people for services rendered. The publication of a scale of salaries deemed fair by a body of nurses numbering almost every woman of distinction in the profession made a great impression on the business men who for the most part manage institutions. Although there are still many laggards, an immediate change for the better was effected all over the kingdom, and it has been our pleasant part to chronicle the efforts which were made to approximate salaries to what was considered reasonable by leaders in the nursing world.

A good deal of water has passed under the bridge during the last two years, and the College now revises its scale in the light of the further depreciation in the value of money, and the further rise in the value of trained nursing. We print the scale in full on p. 373. The salary suggested for matrons of major hospitals is now £500, with an annual increment of £25, graduating downward in accord with the diminishing size of the institution and the lessened responsibilities attaching to the matron's post till it stands at £150 for establishments containing less than twenty-five beds. For assistant-matrons the highest suggested minimum is £150, rising by annual increments of £15 to £225; the lowest for hospitals containing less than 100 beds is £85 to £130. The ward-sisters' minimum stands now at £85 to £120 in the larger institutions; £80 to £90 in the smaller ones; that of the staff nurses, after termination of their agreement, at £60 to £70 irrespective of the number of beds. The suggested scale for probationers is £18, £22, £30, and £40. In every case uniform to be provided or monetary equivalent. The Council deprecate the practice in some institutions of increasing the salary of the probationer out of all proportion to that of the trained nurse. This practice, a policy of despair, has the effect of depreciating the value of the training given, and rousing in the mind of all thoughtful women distrust of a profession which offers so poor a prospect to the trained nurse who has given three years to qualify.

The announcement made by the Council of the College that a superannuation scheme for the profession is under consideration will rouse universal interest. Nothing serious has been done in this direction save by a few hospitals so well situated as to have the means of pensioning officers who have completed long terms of service. The disgrace which attaches to the nursing profession of using the best years of a woman's life and then acquiescing in her being turned adrift unfitted for any other career is still with us. If it can be effaced by a practical measure which all institutions can adopt without undue financial strain, the College will have solved one more of the pressing problems in the profession.

Outside the institution world the recommendations put forward by the College will be welcomed by many. The minimum of £250 for all non-resident posts filled by fully-trained nurses is a pronouncement which should give a lead in the right direction for school nurses, health visitors, and other officials whose prospects are often cramped by municipal and other authorities. A £10 addition for every required special qualification is an admirable feature of this minimum rate, and should clear up many anomalies. We confess ourselves doubtful as to whether the general public will, or, indeed, can, rise to a minimum rate of three and a-half guineas per week for fully-certificated private nurses, boarded and lodged, with laundry in addition. This remains to be seen. We are fully in accord with the dictum that the resident district nurse should receive from £85 to £120.

